# Application of Intelligent Analysis Technology of Football Video Based on Online Target Tracking Algorithm of Motion Characteristics in Football Training

**DOI:** 10.1155/2022/4739712

**Published:** 2022-08-09

**Authors:** Chao Wu, Xin Zhao, Gang Jin

**Affiliations:** ^1^Physical Education Department, University of International Business and Economics, Beijing 100029, China; ^2^Department of International Economy and Trade, School of Humanities and Management, Guangdong Medical University, Guangdong 524023, China; ^3^Physical Education Department, Northeastern University, Shenyang 110819, Liaoning, China

## Abstract

Because the scene of football video is fixed and simple, the events in football video, such as shooting and offside , also have clear semantics. At the same time, they have sufficient domain knowledge and have broad application prospects. The research framework of sports video intelligence analysis is usually regarded as a three-level framework, namely, the low-level feature layer, the middle-level key primitive generation layer, and the high-level event analysis layer. A MF_O2T (Moving Feature Online Target Tracking) algorithm is proposed. First, based on the marked first frame image, this algorithm extracts a set of standardized local images from the target areas of visible and infrared images as target convolution filters, removes the main color of the stadium by using HSV color space nonuniform quantization algorithm, and extracts the histogram of the main color of players in upper and lower blocks. Experimental results show that the algorithm designed in this paper has strong robustness, can better adapt to player tracking in different scenes of football video, and meets the real-time requirements of football training.

## 1. Introduction

In recent years, with the development of science and technology and the improvement of people's requirements for quality of life, the demand for video target detection and tracking technology has become more and more extensive [1]. As one of the favorite sports videos of the audience, the research of detection and tracking algorithms in football match videos has attracted researchers' attention [2]. Video-based target tracking (also known as visual tracking) has always been an important topic and research hotspot. Visual tracking estimates the position, shape, or occupied area of the tracking target in a continuous video image sequence and determines the movement information of the target, such as the moving speed, direction, and trajectory, so as to realize the analysis and understanding of the behavior of the moving target, so as to complete more advanced tasks. People's demand for sports videos is not limited to direct viewing and simple browsing but gradually developing into multifunction and diversification, especially in the detection of specific events (such as wonderful goals) and summary of wonderful clips, hoping to get more information and more detailed services from them [3]. Therefore, in the face of such a huge amount of video data, how to design an effective scientific management scheme and enhance the user experience when consuming video data has become an urgent problem to be solved and optimized.

Although multisensor target tracking is beneficial to target capture, there are still many uncertainties in this algorithm, so developing a reliable and effective multisensor tracking algorithm still needs the joint efforts of a large number of researchers. Seoul National University R&DB Foundation [4] proposes an algorithm of target detection and tracking in football video based on particle filter, which solves the nonlinear and non-Gaussian problems of target detection and tracking in football video. Chen and Huang [5] proposed a target detection and tracking algorithm in football video based on background matching. This algorithm aligns the background images in football video by correlation matching algorithm and combines it with the interframe difference algorithm [6]. However, the detection and tracking results of this algorithm are easily interfered by the outside world, and the detected images are blurred. Li and Chen [7] introduced confrontation learning and generate occlusion masks to simulate occlusion samples for classifier training, so as to improve the classifier's robustness to occlusion. Li and Liu [8] connected the features of the target and the search area to directly return to the position of the target. This method can realize the tracking speed of 100 frames per second on GPU, but the tracking performance is poor [9]. Li et al. [10] directly used the histogram of RGB components to extract the site information, then divided the image to obtain the position of players, used the method of template matching and Kalman filtering to track players, and used histogram back projection to deal with players' occlusion, but the stability of the algorithm is poor. Fajar and Usagawa [11] made use of the images captured by several static cameras placed on the court according to the strategy, generated the observation model based on the data fusion of multicamera coordinates, projected it on the court plane, and generated a multipeak bidirectional probability function, which represents the possible positions of players on the court plane. Liu and Dai [12] put forward that Canny edge extraction is integrated into optical flow field segmentation technology. Experiments show that the proposed method has achieved satisfactory results for single moving target and multiple moving targets, and the real-time performance has been greatly improved [13]. The labeled image blocks are used to train the binary classification network, and good results are obtained, but the training process is complicated.

There are many sports video application scenarios, so there are various intelligent analysis tasks involved. This paper studies the task of automatic statistics of players' running distance based on online target tracking technology and the task of semantic event analysis of football based on group behavior recognition technology. The former designs an online target tracking method that balances performance and efficiency, and then MF_O2T (Moving Feature Online Target Tracking) algorithm scheme is used in football training.

## 2. Research Method

### 2.1. Intelligent Analysis of Football Video

The target tracking algorithm receives various interferences in the actual complex application environment, including background similar interference, changes in lighting conditions, occlusion and other external factors, as well as changes in target attitude. And when the target tracking algorithm is put into practical application, an inevitable real-time problem is also important. It is these problems that make the algorithm research full of difficulties and challenges The basic principle of target tracking algorithm in football game video is as follows: based on the detection results of the target in football game video [14], analyze the external information of the tracked target, calculate the areas with high similarity between two consecutive frames of image information in the video, and record the position and trajectory of the target centroid (center of mass) in football game video [15]. When the target is blocked or lost in tracking, it is necessary to have a certain self-recovery ability. It is not easy to meet the three conditions of real-time detection, accurate detection, and reliability at the same time [16]. At present, the research of target detection and tracking algorithm in football game video encounters some problems, such as complex background interference, occlusion, shadow and balance of real-time, and robustness.

**Figure 1:**
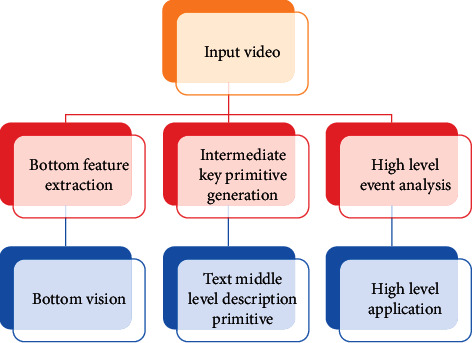
Sports video analysis structure.

The research framework of sports video analysis is usually regarded as a three-level framework, namely, the low-level feature layer, the middle-level key primitive generation layer, and the high-level event analysis layer (see Figure 1).

In sports video analysis, there is a semantic gap between the bottom layer and the top layer. In order to solve this problem, it is necessary to establish an intermediate description layer to bridge the low-level and high-level semantics. Key text elements include scenes and artificially added subtitles, such as “score” and “foul.”

In order to capture the changes of the target (and background) in the tracking process, online visual tracking needs to include an online update mechanism, which constantly updates the appearance model during the tracking process. Common update methods of the appearance model include template update, incremental subspace learning algorithm, and online classifier. How to design a reasonable online update mechanism, which can capture the changes of the target (and background) without causing model degradation, is also a key issue in online visual tracking research. If monitoring all features sounds like a time-consuming task, we can monitor some key features whose data distribution changes may affect the model results. Another solution might be to weight the data. In fact, some algorithms allow you to weigh the importance of input data.

### 2.2. A MF_O2T Method

#### 2.2.1. General Framework of Algorithm

Online target tracking algorithm generally consists of artificial design features and shallow appearance model, aiming at designing fast and robust tracking algorithm by using simple and effective visual features and shallow matching or classification model. In this paper, online target tracking technology is used to solve the task of statistics of players' movement data. The main problem of the algorithm is to match and correlate the existing target trajectories according to the target detection results in video images. Real-time performance of tracking algorithm is an important index for online target tracking tasks in pedestrian monitoring, sports video analysis, and other application scenarios. Obviously, online target tracking method can better adapt to this scenario.

Under the same shot sequence, the scale change of the players' appearance in the far shot is usually small, and the movement track is clear. The players in the middle shot account for a large area in the whole image, and the appearance change is large. The players in the near shot can only see the upper body and the shot duration is short, and the off-site shot has no significance for players tracking. Therefore, the research object of this paper is only the players in the far shot of football video, and the scale is assumed to be unchanged.

In the tracking algorithm, the process of building the weight graph is the process of taking the detected ball as the only node in frame 0, building edges between candidate balls in every two adjacent frames from frame 0 to frame *T*, and assigning weights to these edges and each candidate ball [17]. In the algorithm, in order to track effectively, the ball that has been detected, that is, the only node in frame 0, is given a weight of 1, while the establishment of the edge between it and each node in the first frame in the weight graph and the size of the weight are still based on the distance and similarity between them.

**Figure 2:**
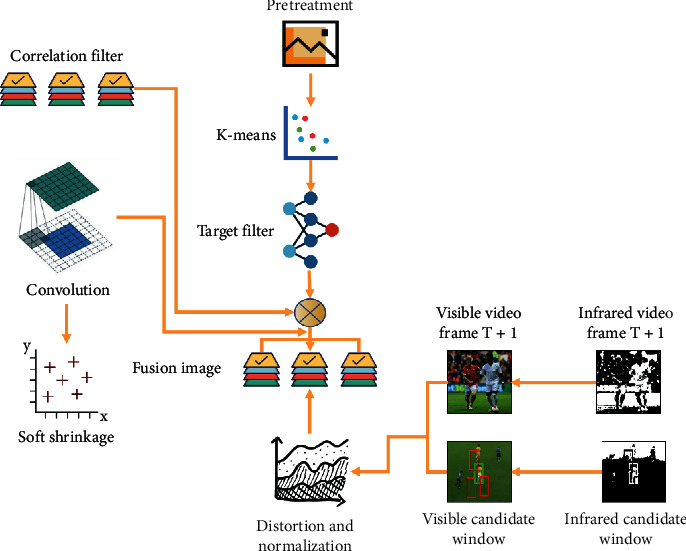
MF_O2T algorithm structure.

MF_O2T algorithm consists of two basic problems, namely feature extraction and tracking algorithm. Feature extraction aims at extracting target features from visible and infrared videos. In this chapter, the algorithm uses CNN (convolutional neural network) without training to extract the target template, and this neural network has only two layers as shown in Figure 2.

First, the construction of relevant candidate windows is used to optimize the feature extraction based only on the target and background, and at the same time, the uncertain input candidate windows are uniformly preprocessed by image warping operation. Then, convolution filters are extracted based on the candidate windows, and local regions corresponding to the input candidate windows are convolved to obtain local selective features. However, the features generated based on different filters can be stacked together to obtain robust features, and this feature is constantly updated, which is conducive to the completion of the algorithm.

#### 2.2.2. Matching Module

In the matching module, cascade matching is used to assign data first, and then global matching is used as a supplement to cascade matching assignment results. In the assignment process, the similarity cost matrix is calculated by the measurement method that meets the requirements, and then the Hungarian algorithm is used to allocate the minimum cost.

For the video with fixed lens, there is no lens motion in the video, so it is more reliable to use target motion features to solve the similarity problem. Mahalanobis distance is scale-independent, which benefits from excluding the influence of dimensions and the interference of correlation between features. Therefore, the square Mahalanobis distance is used here to measure the similarity between the tracking frame and the detection frame. The formula is as follows:

(1) $$\begin{array}{c} d\left(i,j\right)={\left(x_{j}-y_{i}\right)}^{T}C_{i}^{-1}\left(x_{j}-y_{i}\right). \end{array}$$

In the above formula, the *i*-th tracking frame distribution is projected into the measurement space by (*y*_*i*_, *C*_*i*_), and the *j*-th detection frame is represented by *x*_*j*_. This measurement method can greatly save the computing power for extracting apparent features in MF_O2T.

There are many methods of color processing, and the main method is color histogram extraction. For example, in the video of football match, the football field has an obvious main color (green tone), but under different weather and lighting conditions, each stadium will be slightly different. In this paper, we assume that there is a unique main color (green tone) in the football field. The following formula can calculate the main color of football field:

(2) $$\begin{array}{c} Color\;Mean-Osize*\sum_{i=i_{\min}}^{i_{\max}}H\left[i\right]*\frac{I}{\sum_{i=i_{\min}}^{i_{\max}}H\left[i\right]}. \end{array}$$

In this formula, we first convert the color from RGB color space to HSB space and then count the histogram *H*[*i*_min_, *i*_max_] of *H* channel, where *O*size is the quantized size of *H* color histogram.

In long-term target tracking, due to the change of illumination and the shape and posture of the target, the initial target template can no longer reflect the latest features of the target. If the original target template is used continuously, the matching degree will be greatly reduced, resulting in inaccurate target positioning. If the actual size of the target is smaller than the bandwidth of the kernel function, that is, the bandwidth of the kernel function is too large, a large number of background interference pixels will be mixed into the search window [18]. The local convergence of MS (mean shift) iteration will be caused, and the rectangular frame of the target will stay at the local position of the target, resulting in inaccurate positioning.

At the same time, if the algorithm can properly update the scale changes of the target and the feature changes in the target model, it can greatly improve the accuracy of the algorithm location. Based on this, motion detection is introduced for target relocation, scale updating, and template updating. When the size of the target changes little, the scale of the target can be updated only when the size of the target changes obviously. A degree of such target change, measured by relative scale change factor *η*_*h*_, is defined as

(3) $$\begin{array}{c} \eta_{h}=\frac{\left|h-h_{1}\right|}{h}\times 100\%, \end{array}$$

where *h* represents the target bandwidth of the previous frame and *h*_1_ represents the detection reference target bandwidth. Compare *η*_*h*_ with the threshold *σ*_*h*_. If the former is small, keep the original bandwidth; otherwise, update the scale so that *h*=*h*_1_ and general *σ*_*h*_ can take 10%.

After calculating the bandwidth update, the similarity between the candidate template and the target template in the new position $\hat{y}$ of the candidate target obtained by iteration is *ρ*, and the similarity between the target candidate template and the target template in the previous frame is *ρ*_1_.

The relative change of Bhattacharyya coefficient can be expressed by the following formula, and the result *n* is recorded as the relative similarity change.

(4) $$\begin{array}{c} \eta_{\rho }=\frac{\left|\rho_{1}-\rho \right|}{\rho_{1}}\times 100\text{\%}. \end{array}$$

Compare *η*_*ρ*_ with threshold *σ*_*ρ*_. If the former is greater than the latter, it means that the color distribution of the target changes greatly, and the template should not be updated at this time, which may be caused by the occlusion of the target. On the contrary, if the former is smaller than the latter, the change of color distribution is not obvious, and the template should be updated.

**Figure 3:**
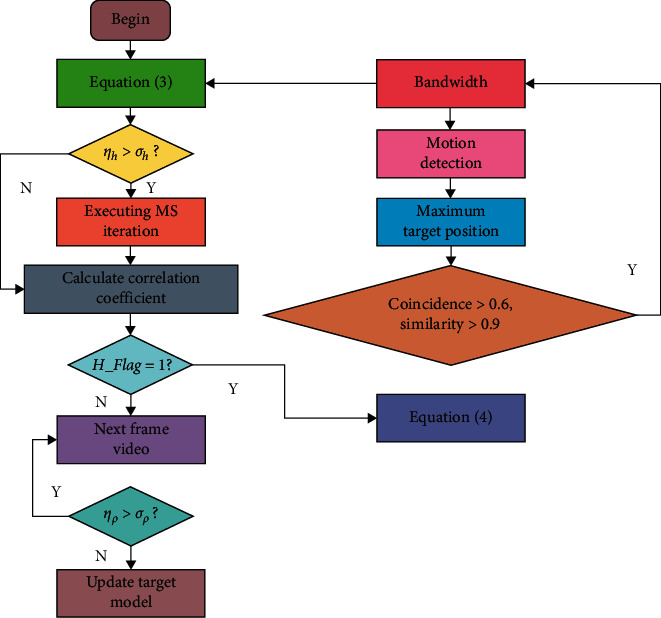
Bandwidth and template updating.

The flow chart of scale update and template update is shown in Figure 3.

#### 2.2.3. Tracking Strategy

When the frame rate is high, the position and size of players between adjacent frames change little. According to this phenomenon, the player with the largest overlapping area in two adjacent frames is considered to be the same player. In order to avoid the error caused by the rapid movement of the camera, the largest overlapping area should not be less than the threshold. The background subtraction method can be used to detect the moving area under the still camera. In order to overcome the time-consuming shortage of the connected region analysis method, the center of gravity shift iteration method is proposed to quickly obtain the moving target of interest. This paper presents a target extraction method based on tracking results. This method combines cpd features with appropriate morphological filtering strategies. The motion under the moving camera is extracted from the Cong tracking results to solve the problem that the target is disturbed by the dynamic background. After processing a frame, each area corresponds to a player, which is assigned its own attributes [19].

The threshold is determined according to the mean value of the local image of the vector field. Then, threshold segmentation is performed on the target image to obtain the initial segmented image, and median filtering and closing operations are performed on the segmented initial image. Kalman filter is used to process the segmented target image, and the centroid position of the target in the shot and the rectangular frame outside the target are selected and tracked.

The candidate window is obtained based on the overlapping rate of the local image generated by the candidate window and the tracking window of the previous frame, in which *m* candidate windows of each category are extracted for each frame image.

(5) $$\begin{array}{c} overlap\;ratio=\frac{Area\left({ROI}_{T-1}\cap {ROI}_{T}\right)}{\left({ROI}_{T-1}\cup {ROI}_{T}\right)}. \end{array}$$

Here, the window of the previous frame is ROI_*T*−1_ and the tracking window is ROI_*T*_.

Normalization method uses the trend and variance of center aggregation to make statistical measurement and then removes outliers to complete the distribution of extended data and increase the uniformity. This is a simple normalization method, which only needs to determine the mean and standard deviation of the input data associated with each input. Set *D*_min_ as the average minus a certain amount of standard deviation. The formula is as follows:

(6) $$\begin{array}{c} D_{\min}=\mu -\sigma_{1}\times \sigma_{2}, \end{array}$$

where *μ* is the mean value of the data and *σ*_1_, *σ*_2_ represents the standard deviation of the selected part of the data, respectively.

As one of the simplest unsupervised learning algorithms to solve clustering problems, K-means has been widely used. This algorithm is mainly a process of classifying a given data set through a number of *k* clusters.

This algorithm means that we associate each sample in the data set with the nearest centroid. When there are no points to be processed, the grouping is completed this time by default. The algorithm aims to minimize the objective function, in which case, we can use the square error function. The function can be written as

(7) $$\begin{array}{c} Loss=\sum_{j=1}^{k}\sum_{i=1}^{m}\left\|1\left\{c^{i}=j\right\}x^{i}-c^{j}\right\|, \end{array}$$

where 1{*c*^*i*^=*j*} represents the discriminant that *x*^*i*^ data are classified into *j* class. If the result is 1, it means it is classified into *j* class; otherwise, it means nothing, so ‖1{*c*^*i*^=*j*}*x*^*i*^ − *c*^*j*^‖ represents the distance between data *x*^*i*^ of *j* class and centroid *c*^*j*^ of this class.

In order to make features more robust to image blurring and appearance changes, a sparse vector is proposed to approximate feature *C*. First, we need to change the feature *C* into a vector *C* ∈ *R*^(*n* − *w*+1)^2^*d*^, and then we use the objective function to obtain the sparse vector *c*′:

(8) $$\begin{array}{c} \underset{c^{\prime }}{\arg\max}\alpha {\left\|c^{\prime }\right\|}_{1}^{1}+\frac{1}{2}{\left\|c^{\prime }-c\right\|}_{2}^{2}. \end{array}$$

According to the above formula, an approximate solution can be obtained by using the soft threshold algorithm [20].

(9) $$\begin{array}{c} c^{\prime }=\text{sig}\left(c\right)\max\left(0,c-\alpha \right), \end{array}$$

where sig(*c*) represents the sign of the acquisition vector *c*.

Based on the assumption that the higher the similarity, the closer the detection scores of training samples should be, the detection scores of training samples can be corrected according to the similarity between training samples. For the *i*-th training sample, use the data correlation part in the online target tracking module to calculate the similarity between it and all the training samples and then get its corrected detection score ${\tilde{s}}_{i}^{\det}$ according to the following formula:

(10) $$\begin{array}{c} {\tilde{s}}_{i}^{\det}=\frac{1}{\sum_{j:s_{ij}\geq 0.7}\exp\left(\beta s_{ij}\right)}\sum_{j:s_{ij}\geq 0.7}\exp\left(\beta s_{ij}\right){\tilde{s}}_{j}^{\det}. \end{array}$$

The cross-camera trajectory matching problem is expressed as the matching problem between local trajectory and target, which is suitable for video scenes with or without overlapping FOV. Each local trajectory is assigned to a unique target, and the proposed constrained nonnegative matrix factorization (rnmf) algorithm is used to calculate the optimal assignment matrix satisfying a set of constraints. The algorithm ensures that the solution meets the matching consistency principle. In addition, the optimal assignment matrix is used, and the information from all local trajectory sets is integrated. We can correct tracking errors caused by occlusion and missed detection in local trajectory sets. A complete and accurate global trajectory is generated for each target in the cross camera. The score of the target trajectory is calculated by the following formula according to the average detection score *s*^tra^ of the historical tracking result of the target and the length *l* of the trajectory:

(11) $$\begin{array}{c} s^{\text{tra}}=s^{\text{his}}\cdot \;\exp\left(-\frac{1}{l}\right). \end{array}$$

The higher the average detection score of historical tracking results, the longer the track length, the higher the target track score and the more reliable the corresponding tracking results.

## 3. Result Analysis

The experimental video of this paper is from the world cup, and the video resolution is 1420 × 976. By studying the network video reports of the football World Cup, this paper summarizes its reporting characteristics and analyzes its development and change and look forward to its future. The research object of this paper mainly focuses on the dissemination and reporting of online video on the football World Cup. In order to evaluate the performance of the algorithm more comprehensively, some statistical data are given in Table 1.

The algorithm chooses the best path in the candidate balls of consecutive *T* frames, and the accuracy of the algorithm will be different if the number of frames is different, that is, *T*. When *T* is small, because the court line or players may be improperly divided in several consecutive frames, and the ball just happens to be blocked or merged, the paths of these court lines or players' small pieces may be more similar to the ball's trajectory, thus prone to false detection in several consecutive frames; when *T* is large, this situation will basically not appear, but the response speed of the algorithm will be slow.

In order to verify the effectiveness of MF_O2T's tracking algorithm, a tracking algorithm based on Kalman prediction is implemented, and the same football game video is tracked for comparative experiments. In the comparative experiment, first, the ball is detected by MF_O2T algorithm, then the motion prediction is made based on Kalman filter, and each target and corresponding features are extracted in the prediction area, and the unique ball is determined by judging the features. The experimental statistics based on Kalman are shown in Table 2.

Comparing Tables 1 and 2, we can see that the recall and accuracy of Table 1 are higher than those of Table 2 on the whole, but the experimental results of Table 1 are based on multiframe image processing, while the experimental results of Table 2 are based on single-frame image processing, so the higher accuracy of MF_O2T algorithm is based on higher time cost.

For MF_O2T algorithm itself, the efficiency and accuracy will be different with different *T*. Therefore, in the process of application, we need to consider the different applications, weigh the efficiency and accuracy, and choose the appropriate algorithm.

**Figure 4:**
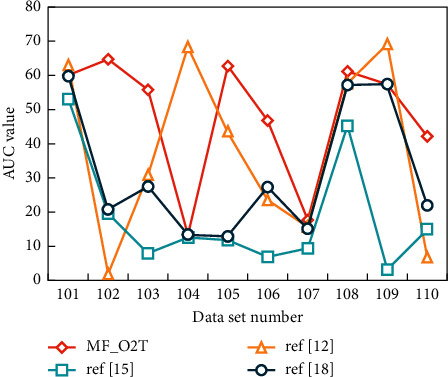
AUC value of tracker in mixed scene.

The data set in the mixed scene contains various possible situations of the target, such as crowding around the players, alternating occlusion of the same team and occlusion of different teams, players falling down, and so on, which directly reflects the robustness of the tracker. The AUC (area under curve) value of the tracker under each data set in this scene is shown in Figure 4.

From the above table, it can be seen that the tracker has low AUC values on many data sets, and MF_O2T algorithm has the best performance on 10 data sets, among which the target players in data set 110 have occlusion and illumination changes but still have not lost their targets. In this scene, the phenomenon of target loss is serious, mainly due to the players in the same team around.

In terms of time performance, the average frame rate of the tracker on all data sets is shown in Table 3:

MF_O2T algorithm is much slower, mainly because particle filtering is used to generate the position candidate set, and each frame needs to requantify the image by HSV color space nonuniform quantization algorithm, calculate the similarity of all particles, and then resample the particles, which takes a lot of time.

**Figure 5:**
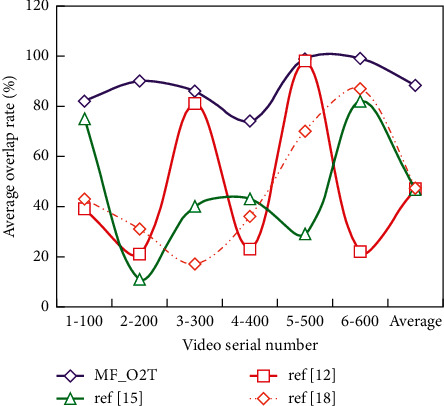
Average overlap ratio comparison.

**Figure 6:**
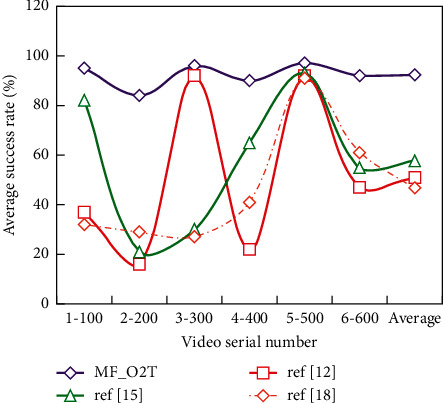
Comparison of average success rate.

Quantitative analysis is the analysis completed by numerical comparison between visible and infrared videos using two evaluation criteria of average overlap rate and average success rate. According to Figures 5 and 6, we can see the experimental results of four algorithms from two aspects of average overlap rate and average success rate.

From the table, we can see that MF_O2T algorithm and ref [15] have the same and the best average central error in video 1–100 mmc1-100, but ref [15] algorithm is poor in average overlap rate and average success rate, mainly because the algorithm is robust to scale changes, so the size of the tracking window is consistent with the label results when there may be little difference in the position of the tracking result window, so a reasonable result is obtained.

In addition, it can be found that MF_O2T algorithm has the best tracking effect in three indexes compared with other algorithms. Although the operation speed of this algorithm is not the fastest among them, the operation speed is within the acceptable range. The way to speed up the algorithm is also simple because now the algorithm is run by CPU. Once CNN is run by GPU, the algorithm time will be greatly shortened.

As a CNN algorithm without training, and the number of layers of the algorithm is only two, this correlation deep learning algorithm can effectively explore the correlation between images and then complete the algorithm based on a correlation weight of each image related to the target. And there is no waste of time for the fusion algorithm, but it is effectively fused because the weight of this fusion method is allocated by infrared light and visible light, and this allocation method is much more efficient than any given model algorithm.

In fact, the weight of correlation tracking algorithm is obtained according to experiments, but there is no way to confirm whether it is appropriate. In fact, this weight can be trained in CNN. However, this method has not been adopted for the time being because of the bad setting of algorithm labels and the influence on algorithm running speed, but in fact, it is a suitable algorithm development direction.

## 4. Conclusion

This paper studies the automatic running distance statistics task based on online target tracking technology and the semantic event analysis task based on group behavior recognition technology. The former designs an online target tracking method that takes into account both performance and efficiency and then applies the MF_O2T (motion feature online target tracking) algorithm scheme to football training. The highlight of the algorithm is that it combines the nontraditional correlation tracking concept and applies it to CNN. Second, this method uses two-layer CNN to supplement the running time. When the detection performance is improved, the tracking accuracy can be improved more effectively than before. In addition, this method achieves a balance between performance and efficiency and is more suitable for real-time application scenarios. Because the background color in the soccer video scene is usually simple, we can consider automatically segmenting all targets in the image in the first frame and performing automatic initialization and online target tracking at the same time.

There are some limitations in this paper. This kind of method does not consider the background information of the target, and the image information has not been well applied. It is simple to automatically segment all objects in the image in the first frame. In the future, it is necessary to supplement the research of automatic initialization and online target tracking. In the future, it is necessary to establish a target model in combination with online learning and then use the model to search the image area with the smallest reconstruction error to complete the target location.

## Figures and Tables

**Table 1:** Experimental statistical data of MF_O2T algorithm.

Video serial number	Soccer	False drop	Recall (%)	Accuracy (%)
1–100	88	10	95.6	90.2
2–200	73	12	87.4	85.3
3–300	42	26	63.6	56.9
4–400	98	2	98.9	99.2
5–500	93	7	94.5	74.5
6–600	87	5	95.1	79.8

**Table 2:** Experimental statistical data based on Kalman algorithm.

Video serial number	Soccer	False drop	Recall (%)	Accuracy (%)
1–100	86	12	94.1	87.1
2–200	79	11	90.3	88.3
3–300	33	21	51.2	97.1
4–400	81	3	83.6	90.2
5–500	89	9	89.9	76.3
6–600	70	5	77.8	95.2

**Table 3:** Tracker average frame rate.

Tracker	Average frame rate (FPS)
Ref [12]	26.31
Ref [15]	69.74
Ref [18]	156.38
MF_O2T	30.44

## Data Availability

The data used to support the findings of this study are available from the corresponding author upon request.
